# Direct Observation of Structural Unraveling in PABPN1 Polyalanine Expansions

**DOI:** 10.1021/acsbiomedchemau.6c00065

**Published:** 2026-08-06

**Authors:** Rosa Antón, Javier Oroz

**Affiliations:** Instituto de Química Física Blas Cabrera (IQF), CSIC. Serrano 119, E-28006 Madrid, Spain

**Keywords:** polyalanine, PABPN1, polymorphism, real-time NMR, OPMD, homorepeat

## Abstract

Above-threshold homorepeat expansions promote alternative conformations and irreversible aggregation, driving the malfunction of numerous disease-associated proteins. A detailed understanding of the structural remodeling events in pathogenic mutants enables the identification of key elements determining aberrant structure formation. Here, we describe in high resolution the structural transitions in Poly­(A) Binding Protein Nuclear 1 (PABPN1), in which polyalanine expansions beyond a +1 single additional residue cause Oculopharyngeal Muscular Dystrophy (OPMD). PABPN1’s polyalanine tract in its wild-type (containing 10 consecutive alanine residues) and +1 and +8 variants populate alternative polymorphs, with α-helical conformations being increasingly favored and rigid upon expansion. Spontaneous slow unfolding was observed across variants in real time by Nuclear Magnetic Resonance (NMR) spectroscopy, indicating that increased dynamics at the C-terminal end of the polyalanine helix emerges as an early unfolding event associated with functional impairment. Our data help to define key mechanistic features in pathogenic misfunction induced by homorepeat expansions and identify specific structural motifs as potential targets for fragment-based binders to restore protein functionality in polyalanine expansion disorders.

## Graphical abstract

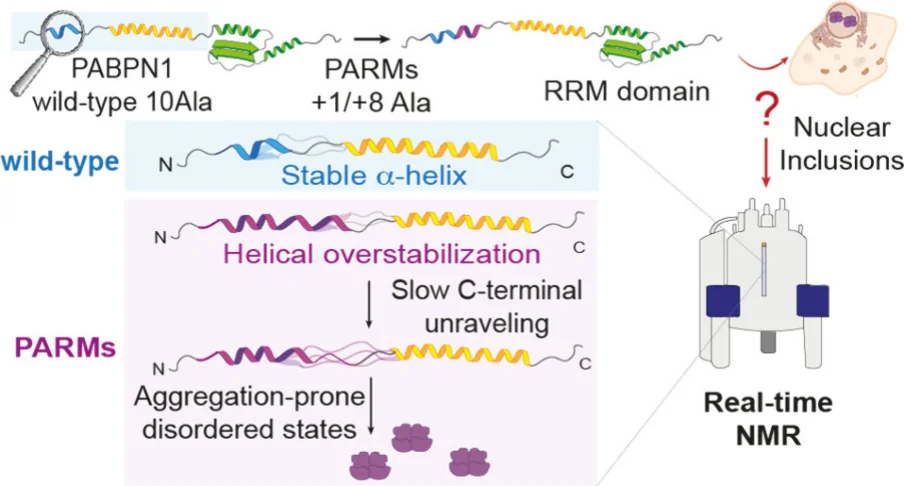

Polyalanine-containing transcription factors and RNA-binding proteins constitute a prominent class of human proteins in which short homorepeats can undergo pathogenic expansion through mismatched DNA recombination.^1^ When the length of such polyalanine repeat mutations (PARMs) surpasses critical sequence-dependent thresholds, the expanded proteins often misfold, mislocalize, or acquire aberrant gain-of-function properties, collectively leading to nine known human developmental and neuromuscular disorders.^2–4^ For most PARM-associated diseases, the appearance of nuclear or cytoplasmic inclusions composed of expanded proteins correlates strongly with disease onset and severity.^5–12^ Therefore, understanding the structural basis of this intriguing propensity for aggregation in PARMs is fundamental for designing effective strategies aimed at rescuing protein functionality in expanded proteins. We recently demonstrated, using PHOX2B—which harbors the longest polyalanine tract in mammals—that alanine expansions induce nascent disordered states within otherwise stable α-helical homorepeats.^13^ These metastable, partially unravelled conformers act as nucleation points for the formation of irreversible, solid-like condensates, a mechanism that underlies the molecular pathology of Central Congenital Hypoventilation Syndrome (CCHS).^13^ Considering the relevance of the neighboring effects in capping the conformational stability of homorepeats,^14^ we sought to determine whether the increased flexibility of the polyalanine α-helix is a general mechanism triggering aberrant aggregation across PARMs. Here, we have characterized the structural dynamics of the RNA-processing factor PABPN1, which shows pathogenic PARMs in OPMD, an incurable rare disorder with a worldwide prevalence of 1–9/100,000.

Human PABPN1 contains 10 consecutive alanine residues immediately following the initial Met, and expansions from +1 (11 consecutive alanine residues) to +8 (18 alanines) show strong penetrance in the disease.^15^ Whereas wild-type PABPN1 forms benign, manageable nuclear inclusions that support RNA processing, expanded PABPN1 forms pathogenic aggregates in muscle cells,^16^ impacting nuclear morphology and causing protein loss-of-function.^17^ RNA binding is required for PABPN1 condensation into nuclear speckles, but alanine expansion leads to its insoluble aggregation and the sequestration of essential components of the RNA processing machinery.^18^ This pathogenic mechanism resembles those described for additional transcription factors affected by polyalanine expansions in hereditary synpolydactyly^11^ and CCHS.^13^ Therefore, prevalent structural alterations induced by PARMs may underlie these mechanistic commonalities. Building on our observations in the structural malleability of polyalanine-expanded PHOX2B,^13^ we employed NMR spectroscopy to understand the structural impact of PARMs in PABPN1. Taking advantage of the peculiar N-terminal location of the polyalanine segment in PABPN1 (Figure 1A), we have monitored at single-residue resolution the evolution of the conformational transitions explored by the polyalanine tracts, in the wild-type and pathogenic PABPN1 variants. Secondary chemical shift analysis across the region covering residues 1–170 from human PABPN1 including 10, 11, and 18 consecutive alanine residues shows an increased propensity to populate α-helical conformations in the expanded mutants (Figure 1B). Examination of the ^15^N-HSQC spectra of the different proteins at 25 °C reveals multiple sets of signals for the polyalanine segments, indicative of alternative polymorphism in these particular regions (Figure 1C, Supplementary Figures 1, 2).

**Figure 1:**
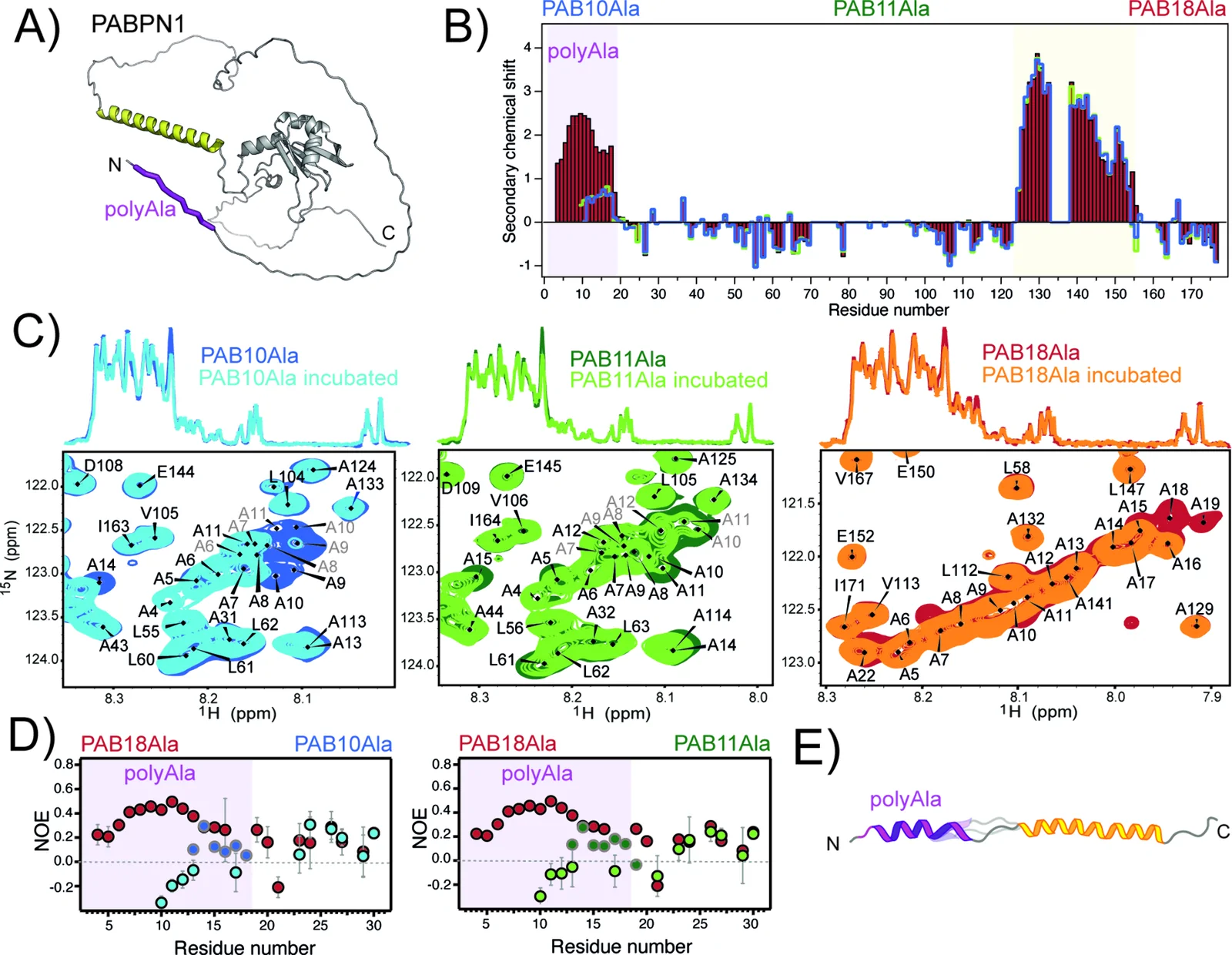
Structural exchange in PABPN1 polyalanine homorepeats captured in real time by NMR. (A) AlphaFold structural prediction for human PABPN1 (Uniprot code Q86U42). The polyalanine stretch is colored in magenta and highlighted in ribbon representation. The α-helix located in residues 118–149 is colored yellow for comparison. (B) NMR-based secondary chemical shifts for the 10, 11, and 18 alanine PABPN1 (termed PAB10Ala, PAB11Ala and PAB18Ala for simplicity, colors are indicated). To help with visual interpretation, the data were aligned to match the 18-alanine plot. In this plot, positive values indicate a tendency to populate α-helical conformations. The two helical segments contained in the proteins are limited in magenta (based on the 18-alanine sequence) and yellow, respectively. (C) Detailed ^15^N-HSQC regions for the polyalanine segments of the different PABPN1 constructs, indicated in colors. In the spectra, gray labels correspond to alternative alanine moieties. Spectra were overlaid with those resulting from incubation at 25 °C, displayed in lighter colors for visual comparison. In particular, PABPN1 containing a 10-alanine stretch was incubated for 37 h, PAB11Ala incubated for 27 h, and PAB18Ala for 16 h. Note that signals in gray disappear upon incubation. The higher helical content for PAB18Ala correlates with a different array of signals in the spectra, and upon incubation, crosspeaks corresponding to the terminal alanine residues (Ala18, Ala19), clearly disappear. Above the spectra, the 1D projections for the HSQC spectra at the corresponding incubation times are overlaid, indicating that proteins remain soluble and that changes in the spectra correspond to structural alterations. (D) Overlay of the heteronuclear NOEs for fresh PABPN1 proteins focused on the N-terminal region, for comparison. For PAB10Ala and PAB11Ala, the two sets of signals are indicated in colors related to those in the HSQCs (panel C). Signals circled in gray correspond to gray labels in the HSQCs, which consistently show higher rigidity (higher NOE values). Values were aligned onto PAB18Ala, for comparison. Polyalanine α-helical region is delimited in magenta. Errors were calculated based on the NMR S/N ratio. Note that Ala18 and Ala19 for PAB18Ala disappeared during the acquisition time. (E) Cartoon representation of the PABPN1 segments characterized by NMR, showing the terminal unraveling of the polyalanine α-helix.

While the overall signal intensity in the HSQC spectra was comparable upon incubation indicating lack of protein aggregation during the acquisition time, time-dependent changes were observed in the chemical shifts of the polyalanine segment for all proteins (Figure 1C). In particular, the alanine residues located at the C-terminus of the tract, in addition to immediate neighboring residues (Supplementary Figure 3), underwent slow interconversion on the NMR time scale. In PHOX2B, polyalanine expansions showed slow unraveling of the α-helical homorepeats toward disordered conformations, which trigger irreversible aggregation.^13^ Heteronuclear (^1^H)-^15^N NOEs, which report on ps-ns backbone dynamics and inform on backbone rigidity, confirmed that the two sets of alanine signals within the tract in the PABPN1 variants populate different dynamic regimes, indicative of alternative degrees of flexibility (Figure 1D, Supplementary Figure 4). The population exhibiting higher rigidity (in gray in Figures 1C, D) displayed chemical shifts consistent with α-helical conformations, whereas the more dynamic population could be attributed to partially disordered structures. Overall, data reveal a slow terminal unraveling of the helical homorepeats in PABPN1 (Figure 1E).

A recent study confirmed the transient, disordered states in the PHOX2B polyalanine segment by combining experimental chemical shifts with machine learning models and simulations.^19^ Through maximum entropy reweighting approximations, the method enables to accurately weight the ensemble populations, helping to redefine the polymorphic conformational landscape.^19^ Although signal intensity in the NMR spectra correlates with abundance of conformational states, estimating the populations of particular unfolded conformers based on backbone amide crosspeak intensity can be problematic due to the high amide exchange in the NMR experimental conditions.^20^ Line width in ^13^C-detected experiments is less affected by chemical exchange kinetics and therefore may better reflect the abundance of particular conformational states. CACO and CON experiments were used to describe what particular structures are explored by the homorepeats and the impact of the PARMs in these particular rearrangements (Figure 2, Supplementary Figure 5). CACO experiments confirmed the existence of the double population of conformers in PAB10Ala and PAB11Ala, with the population showing the highest backbone rigidity (Figure 1D) correlating with a higher content in α-helical structure, as determined by the direct observation of Cα chemical shifts (Figure 2A–D). These experiments also enabled to monitor the increase in helicity toward the terminal end induced by the single alanine addition in PAB11Ala, illustrating how even minimal sequence extension modulates local structural bias (Figure 2D). Remarkably, these metastable structures, with a higher helical content and rigidity, showed slow structural unraveling and exchanged into disordered conformations, with a faster rate in PAB11Ala (Figures 2B,D).

**Figure 2:**
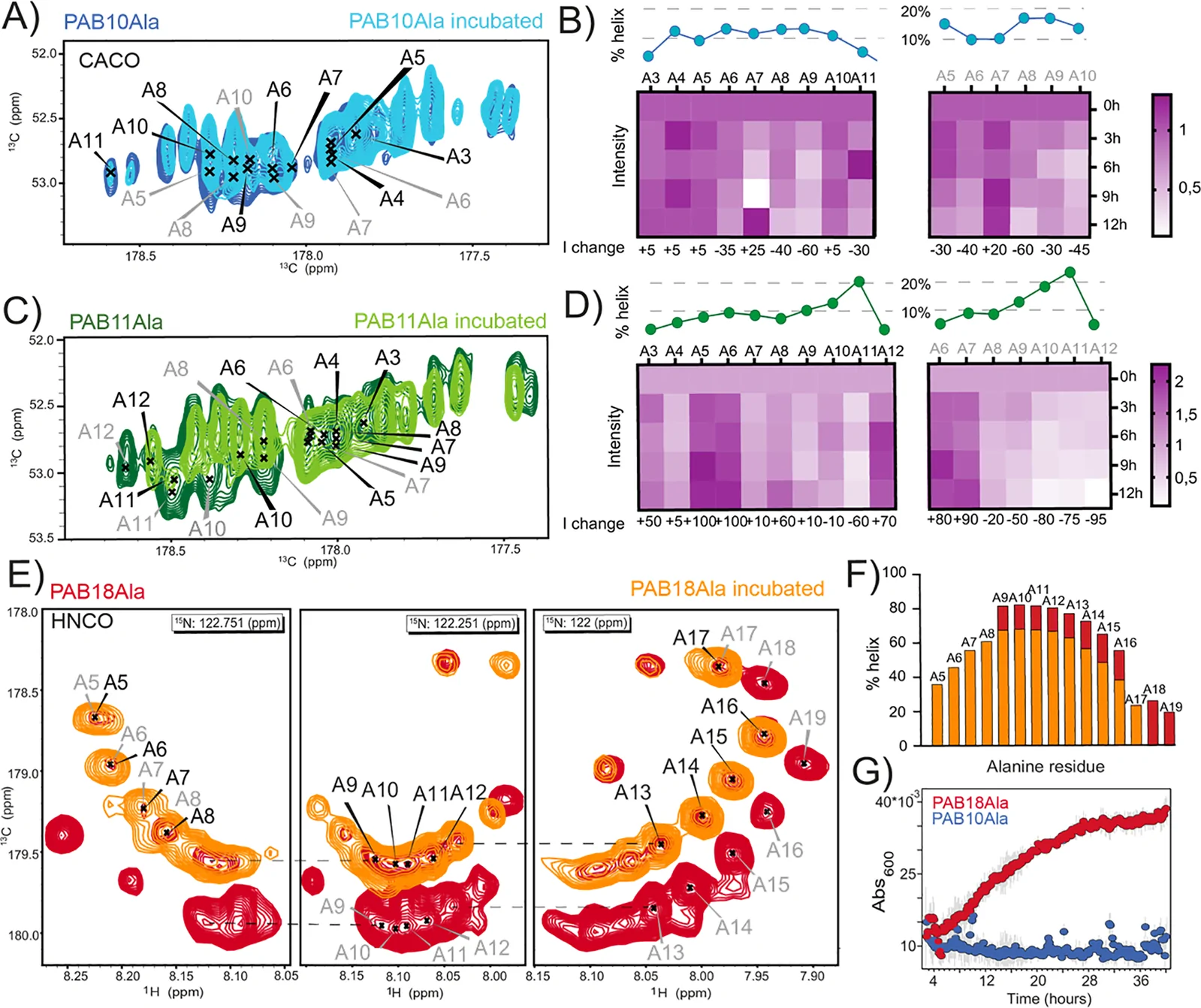
Enhanced flexibility in expanded PABPN1 contributes to condensation. (A) CACO spectra for PAB10Ala focused on the polyalanine segment. Assignments are indicated and follow the same logic as in Figure 1. Overlaid is the CACO spectra of PAB10Ala after 12 h of incubation at 25 °C (in lighter color). (B) Heatmap analysis of the normalized (relative to the intensity at time zero) CACO signal intensity for the individual alanine moieties from the polyalanine segment at the indicated incubation times. Alternative conformations (gray labels) are displayed on the right. On top, the helical propensity for the particular alanine residue is indicated, as calculated with the Cα chemical shifts from the CACO spectra. The alternative population (in gray) shows a higher propensity to adopt α-helical structures. (C) Overlaid CACO spectra for PAB11Ala after 12 h of incubation at 25 °C. (D) Heatmap analysis of the time-dependent decay in signal intensity in PAB11Ala CACO spectra, revealing a significant decrease at the terminal end for the alternative, more structured conformation (box labeled in gray). Normalization scale is represented on the right. At the bottom of the plots (B, D), the change in intensity between the first and last time points per residue is represented. (E) HNCO projections for PAB18Ala, focused on the polyalanine segment. Red spectra correspond to a fresh sample, while the orange spectra correspond to a PAB18Ala sample incubated for 60 h at 25 °C. Assignments are indicated. Broken lines represent correlations between the projections. Unassigned crosspeaks in the spectra correspond to additional residues. (F) Estimation of α-helical content of the individual Ala residues obtained from the HNCO spectra. Colors match those in panel E. Terminal residues (Ala 18, 19) were not assigned in the spectra for incubated PAB18Ala (orange). (G) Turbidity at 25 °C for 0,2 mM PAB18Ala (red) and PAB10Ala (blue) as measured by Absorbance at 600 nm. Increase in turbidity corresponds to aggregate formation.^13,16,18^

PAB18Ala has a higher aggregation propensity, leading to decreased spectral quality at ^13^C-detected experiments at identical protein concentrations (Supplementary Figure 5). Nevertheless, HNCO spectra revealed an intricate spectral pattern for the polyalanine segment in PAB18Ala, which evolved upon incubation (Figure 2E). In particular, nascent crosspeaks appeared at upfield ^13^C chemical shifts, associated with states with lower α-helical propensity. These disordered structures became prevalent over time reducing the population of helical conformers, which was particularly evident for the terminal alanine residues (Figure 2F). Overall, data indicate that the metastable α-helical structures promoted by polyalanine stacking in PABPN1 unravel in solution along its C-terminus (Figure 1E). PARMs increase helicity, but the more rigid helical structures rapidly collapse at the end of the polyalanine tract, reflecting extensive helix destabilization and giving rise to disordered structures following a tensioned helix model (Figures 2D,F). Considering that structural disorder triggered by PARMs is coupled to phase transitions in PHOX2B,^13^ we confirmed that expanded PABPN1 shows a higher propensity to aggregate (Figure 2G), which could be attributed to an increase in disordered conformations.

Increase in polyglutamine and polyalanine length has been correlated to predominance of α-helical states.^13,21,22^ While capping interactions are fundamental stabilizing helical conformations in polyglutamine expansions,^14^ the coexistence of alternative metamorphs in steady state has been directly observed now for two polyalanine expanded proteins, PHOX2B^13^ and PABPN1. Inherent flexibility of flanking segments is essential for structural malleability in homorepeats,^1^ and a close inspection of the sequences of human proteins where pathogenic PARMS are located reveals an abundance of short, nonstructuring residues flanking the polyalanine stretches (Figure 3). Indeed, deep sequence analysis of the human proteome identified glycine and proline as the most abundant amino acids flanking polyalanine stretches.^23^ In particular, the presence of Gly in position +1 increases the propensity to adopt nonhelical conformations,^23^ restraining the helical propensity of PARMs. This proximal flexibility may enhance the ability of the polyalanine segments to adopt multiple structural rearrangements as a multiplexed adaptor to bind a multitude of partners.^1^ However, our observations indicate that excessive disorder, as induced by PARMs, may be detrimental, triggering irreversible aggregation.

**Figure 3:**
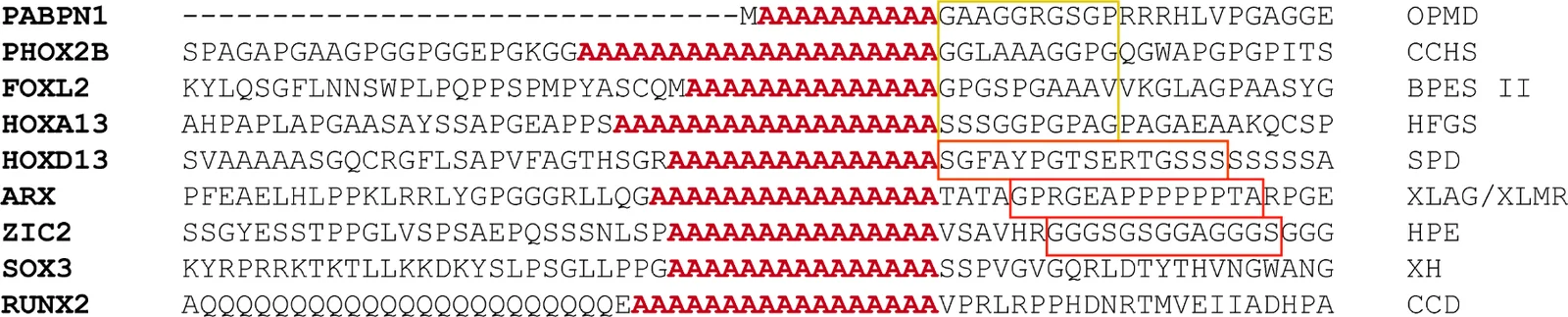
Sequence analysis of the polyalanine tracts locating pathological PARMS. The yellow box maps the GP-rich motif following the polyalanine segment in PABPN1, PHOX2B, FOXL2 and HOXA13. Red boxes map downstream elements in HOXD13, ARX and ZIC2 rich in Gly, Pro and Ser. SOX3 and RUNX2 appear to contain unrelated sequence motifs at the C-terminus of the polyalanine segment. Polyalanine segments are highlighted in bold red. Disease abbreviations correspond to OPMD = Oculopharyngeal Muscular Dystrophy; CCHS = Congenital Central Hypoventilation Syndrome; BPES II = Blepharophinosis, ptosis and epicanthus inversus syndrome Type II; HFGS = Hand-Foot-Genital Syndrome; SPD = Synpolydactyly; XLAG = X-linked lissencephaly with abnormal genitalia; XLMR = X-linked mental retardation; HPE = Holoprosencephaly; XH or PHPH = X-linked panhypopituitarism; CCD = Cleidocranial Dysplasia.

Nine developmental and neurodegenerative disorders are caused largely by expansions of polyalanine segments beyond defined thresholds (Figure 3), which collectively affect more than 100,00 people worldwide.^2–4^ Aggregation triggered by PARMs does not follow canonical amyloid pathways^24^ and, considering the significant propensity of alanine to adopt α-helical structures, the prevailing assumption has been that PARMs aggregate via coiled-coil multimerization.^23–25^ Through high-resolution NMR measurements, we have determined, previously in PHOX2B^13^ and now in PABPN1, that irreversible aggregation is caused by an increase in disorder in the polyalanine segment. PABPN1 represents an intriguing case due to its inherent propensity to form nuclear inclusions,^16^ the N-terminal location of the polyalanine tract, and the fact that a + 1 Ala expansion causes protein malfunction. Real-time NMR observations enable the location of the early unraveling events in PABPN1 on the C-terminal end of polyalanine α-helix, probably contributed by a Gly insertion at the nearby position (Figure 3). Strong conservation of small, flexible, or nonstructuring residues adjacent to polyalanine segments opens the door to establishing analogous mechanisms as responsible for loss-of-function in additional proteins affected by pathogenic PARMs. Therefore, targeting these particular sequence motifs with synthetic α-helical stabilizers emerges as an attractive strategy to recover protein functionality in pathogenic polyalanine expansions. Based on its exquisite detection of nascent polymorphs, NMR spectroscopy is ideally positioned to screen for structure-based binders that could inhibit the noxious conformational exchange observed in pathogenic PARMs.

## Supplementary Material



## Notes

Five supplementary figures with details on full assigned spectra, sequence alignments, conformational exchange and dynamics of PABPN1 variants, in addition to Experimental Details, materials and methods (PDF)

## References

[1] Chavali S., Singh A. K., Santhanam B., Babu M. M. (2020). Amino acid homorepeats
in proteins. Nat. Rev. Chem..

[2] Cummings C. J., Zoghbi H. Y. (2000). Fourteen and counting: unraveling
trinucleotide repeat
diseases. Hum. Mol. Genet..

[3] Bernacki J. P., Murphy R. M. (2011). Length-dependent aggregation of uninterrupted polyalanine
peptides. Biochemistry.

[4] Messaed C., Rouleau G. A. (2009). Molecular mechanisms underlying polyalanine diseases. Neurobiol. Dis..

[5] Trochet D., Hong S. J., Lim J. K., Brunet J. F., Munnich A., Kim K. S., Lyonnet S., Goridis C., Amiel J. (2005). Molecular
consequences of PHOX2B missense, frameshift and alanine expansion
mutations leading to autonomic dysfunction. Hum. Mol. Genet..

[6] Shoubridge C., Cloosterman D., Parkinson-Lawerence E., Brooks D., Gécz J. (2007). Molecular
pathology of expanded polyalanine tract mutations in the Aristaless-related
homeobox gene. Genomics.

[7] Utsch B., McCabe C. D., Galbraith K., Gonzalez R., Born M., Dötsch J., Ludwig M., Reutter H., Innis J. W. (2007). Molecular
characterization of HOXA13 polyalanine expansion proteins in hand-foot-genital
syndrome. Am. J. Med. Genet A.

[8] Wong J., Farlie P., Holbert S., Lockhart P., Thomas P. Q. (2007). Polyalanine
expansion mutations in the X-linked hypopituitarism gene SOX3 result
in aggresome formation and impaired transactivation. Front Biosci.

[9] Fan J., Zhou Y., Huang X., Zhang L., Yao Y., Song X., Chen J., Hu J., Ge S., Song H., Fan X. (2012). The combination of
polyalanine expansion
mutation and a novel missense substitution in transcription factor
FOXL2 leads to different ovarian phenotypes in blepharophimosis-ptosis-epicanthus
inversus syndrome (BPES) patients. Hum. Reprod..

[10] Pelassa I., Corà D., Cesano F., Monje F. J., Montarolo P. G., Fiumara F. (2014). Association of polyalanine and polyglutamine coiled
coils mediates expansion disease-related protein aggregation and dysfunction. Hum. Mol. Genet..

[11] Basu S., Mackowiak S. D., Niskanen H., Knezevic D., Asimi V., Grosswendt S., Geertsema H., Ali S., Jerković I., Ewers H., Mundlos S., Meissner A., Ibrahim D. M., Hnisz D. (2020). Unblending of Transcriptional Condensates in Human Repeat Expansion
Disease. Cell.

[12] Roth F., Dhiab J., Boulinguiez A., Mouigni H. R., Lassche S., Negroni E., Muraine L., Marhic A., Oliver A., Lainé J., Rouche A., O’Ferrall E. K., van Engelen B., Ottenheijm C., Greif H., Blumen S., Lacau St
Guily J., Perie S., Butler-Browne G., Mouly V., Trollet C. (2022). Assessment of PABPN1 nuclear inclusions
on a large cohort of patients and in a human xenograft model of oculopharyngeal
muscular dystrophy. Acta Neuropathol..

[13] Antón R., Treviño M. Á., Pantoja-Uceda D., Félix S., Babu M., Cabrita E. J., Zweckstetter M., Tinnefeld P., Vera A. M., Oroz J. (2024). Alternative low-populated
conformations prompt phase transitions in polyalanine repeat expansions. Nat. Commun..

[14] Escobedo A., Topal B., Kunze M. B. A., Aranda J., Chiesa G., Mungianu D., Bernardo-Seisdedos G., Eftekharzadeh B., Gairí M., Pierattelli R., Felli I. C., Diercks T., Millet O., García J., Orozco M., Crehuet R., Lindorff-Larsen K., Salvatella X. (2019). Side chain to main chain hydrogen
bonds stabilize a polyglutamine helix in a transcription factor. Nat. Commun..

[15] Brais B., Bouchard J. P., Xie Y. G., Rochefort D. L., Chrétien N., Tomé F. M., Lafrenière R. G., Rommens J. M., Uyama E., Nohira O., Blumen S., Korczyn A. D., Heutink P., Mathieu J., Duranceau A., Codère F., Fardeau M., Rouleau G. A. (1998). Short GCG
expansions
in the PABP2 gene cause oculopharyngeal muscular dystrophy. Nat. Genet..

[16] Mallon S. D., Bos E., Sheikhhassani V., Shademan M., Voortman L. M., Mashaghi A., Sharp T. H., Raz V. (2025). Polyalanine Expansion
in PABPN1 Alters the Structure and Dynamics of Its Nuclear Aggregates
in Differentiated Muscle Cells. FASEB J..

[17] Mezzell A. T., Zhang Y., Perez A. M., Vest K. E. (2026). Oculopharyngeal
muscular dystrophy (OPMD) associated alanine expansion impairs the
function of the nuclear polyadenosine RNA binding protein PABPN1 as
revealed by proximity labeling and comparative proteomics. PLoS Genet..

[18] Guan W. L., Jiang L. L., Yin X. F., Hu H. Y. (2023). PABPN1
aggregation
is driven by Ala expansion and poly­(A)-RNA binding, leading to CFIm25
sequestration that impairs alternative polyadenylation. J. Biol. Chem..

[19] Jeon J., Yang W., Park S., Kim J. H., Lee Y. H., Yu W. (2026). Accurate conformational ensembles of intrinsically disordered proteins
using reweighting based on NMR chemical shifts. Proc. Natl. Acad. Sci. U. S. A..

[20] Dreydoppel M., Balbach J., Weininger U. (2022). Monitoring
protein unfolding transitions
by NMR-spectroscopy. J. Biomol NMR.

[21] Bravo-Arredondo J. M., Kegulian N. C., Schmidt T., Pandey N. K., Situ A. J., Ulmer T. S., Langen R. (2018). The folding
equilibrium of huntingtin
exon 1 monomer depends on its polyglutamine tract. J. Biol. Chem..

[22] Elena-Real C. A., Sagar A., Urbanek A., Popovic M., Morató A., Estaña A., Fournet A., Doucet C., Lund X. L., Shi Z. D., Costa L., Thureau A., Allemand F., Swenson R. E., Milhiet P. E., Crehuet R., Barducci A., Cortés J., Sinnaeve D., Sibille N., Bernadó P. (2023). The structure
of pathogenic huntingtin exon 1 defines the bases of its aggregation
propensity. Nat. Struct Mol. Biol..

[23] Mier P., Elena-Real C. A., Cortés J., Bernadó P., Andrade-Navarro M. A. (2022). The sequence
context in poly-alanine regions: structure,
function and conservation. Bioinformatics.

[24] Polling S., Ormsby A. R., Wood R. J., Lee K., Shoubridge C., Hughes J. N., Thomas P. Q., Griffin M. D., Hill A. F., Bowden Q., Böcking T., Hatters D. M. (2015). Polyalanine expansions
drive a shift into α-helical clusters without amyloid-fibril
formation. Nat. Struct Mol. Biol..

[25] Pelassa I., Corà D., Cesano F., Monje F. J., Montarolo P. G., Fiumara F. (2014). Association of polyalanine and polyglutamine coiled
coils mediates expansion disease-related protein aggregation and dysfunction. Hum. Mol. Genet..

