# Supplementary material for: Direct Observation of Structural Unraveling in PABPN1 Polyalanine Expansions
Source: ACS Bio Med Chem Au. 2026 Aug 6;6(5):645–50. doi: 10.1021/acsbiomedchemau.6c00065 (PMC13692728; doi:10.1021/acsbiomedchemau.6c00065)
Supplement: Supplementary file 1 [file bg6c00065_si_001.pdf]

Direct Observation of Structural Unraveling In PABPN1 Polyalanine Expansions

Rosa Antón*, Javier Oroz*

Instituto de Química Física Blas Cabrera (IQF), CSIC. Serrano 119, E-28006 Madrid, Spain.
E-mail: ranton@iqf.csic.es; joroz@iqf.csic.es

Supporting Information

Supplementary Figures

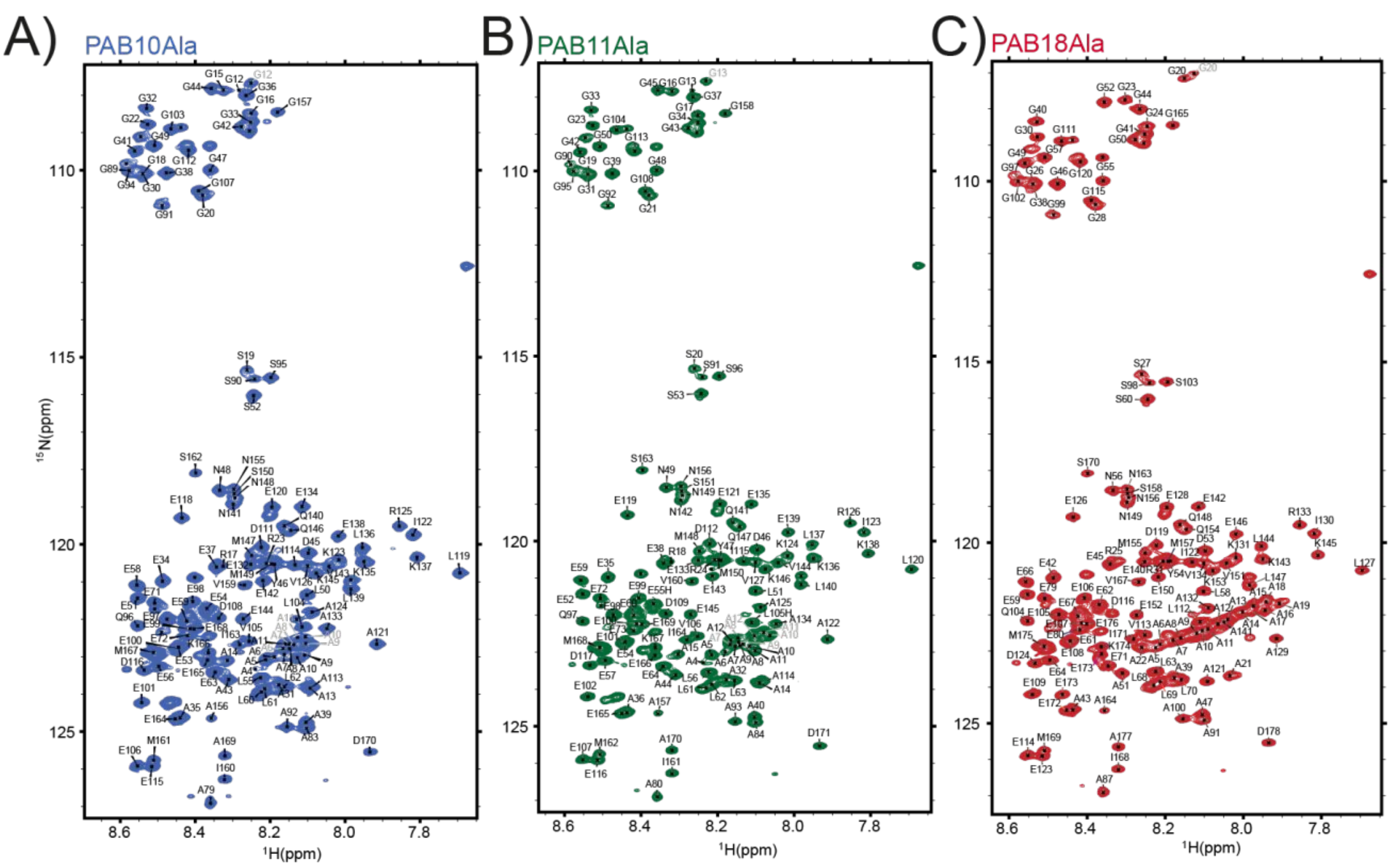

Supplementary Figure 1. Assigned ^{15}N-HSQC of PAB10Ala (blue), PAB11Ala (green) and PAB18Ala (red) at 25 ºC. Assignments in grey correspond to those described in **Fig. 1C.**

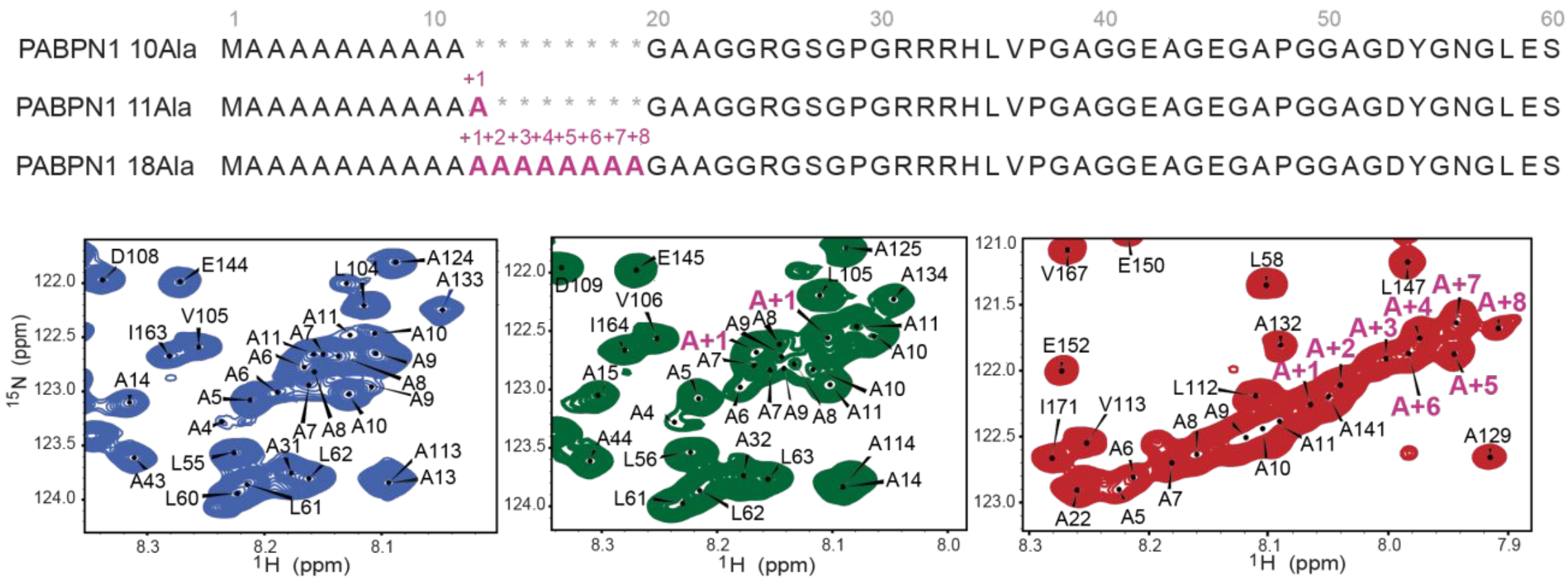

Supplementary Figure 2. Sequence alignment of PABPN1 variants under study and representative HSQC spectra. Sequences are aligned on top, and the additional alanines are numbered accordingly. On the bottom, HSQC spectra for fresh samples are shown, where blue corresponds to Pab10Ala, green to Pab11Ala, and red to Pab18Ala. Residues are assigned, and the additional alanines are numbered and labeled in magenta.

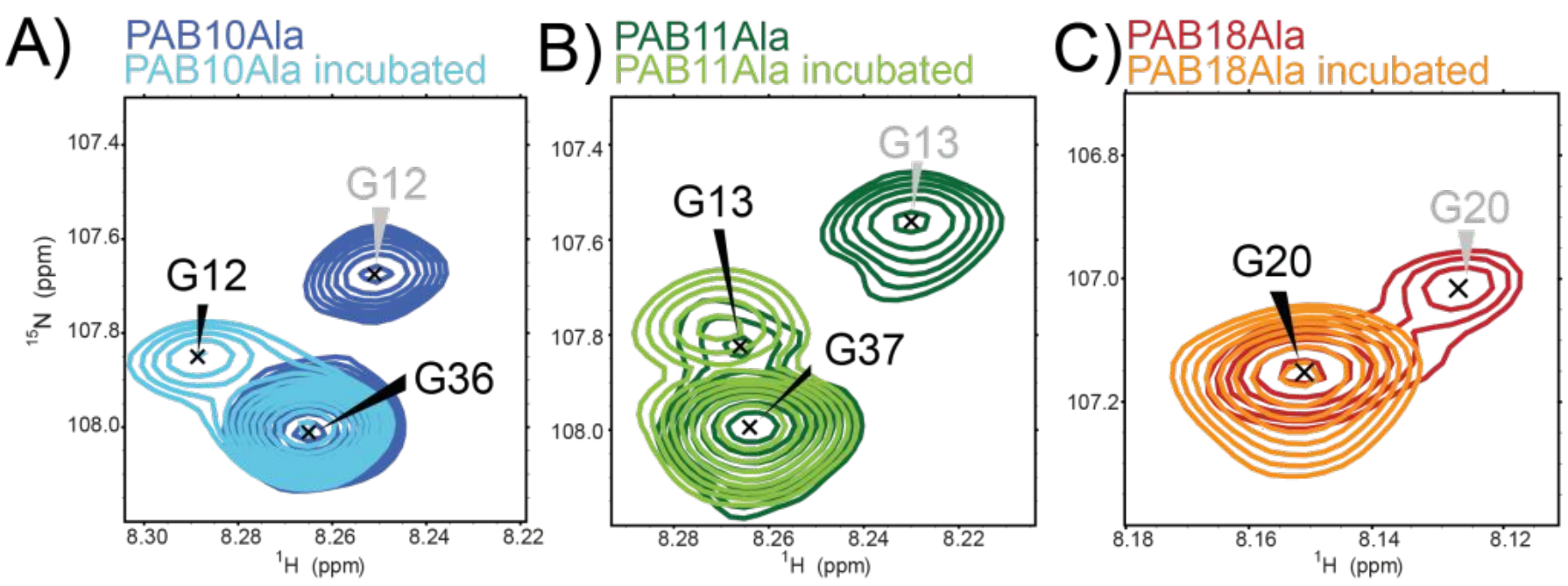

Supplementary Figure 3. Conformational exchange of the Gly residue neighbouring the polyalanine tract in PABPN1. Details of the ^{15}N-HSQC spectra of PAB10Ala (blue), PAB11Ala (green), and PAB18Ala (red) focused on Gly exchange upon incubation at 25 ºC. Lighter colors correspond to those detailed in **Fig. 1C.** Assignments in grey correspond to those described in **Fig. 1C** and in **Supplementary Figure 1.**

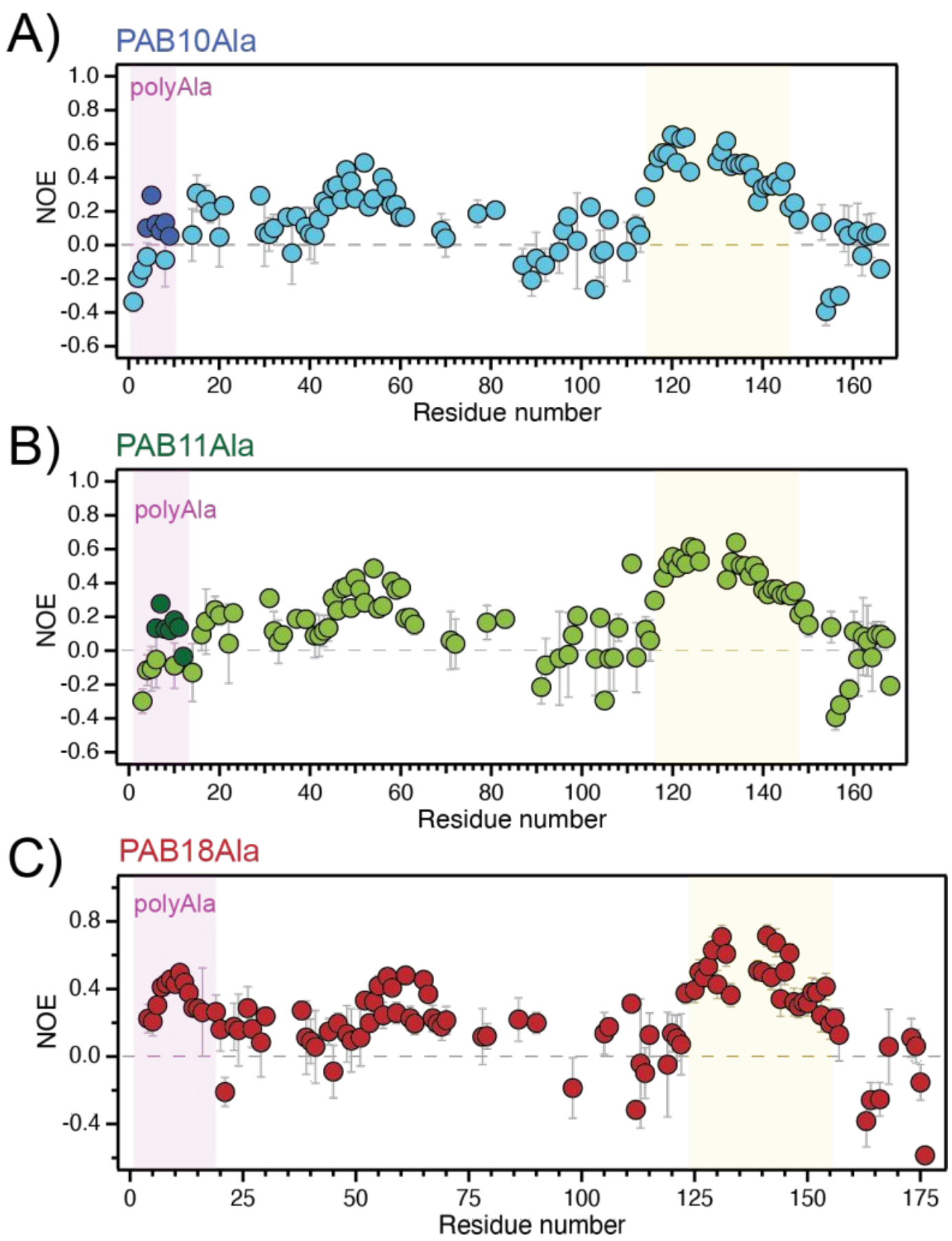

Supplementary Figure 4. ^{15}N-heteronuclear NOEs showing backbone dynamics for PAB10Ala (blue), PAB11Ala (green), and PAB18Ala (red) at 25 ºC. Polyalanine fragment is highlighted in magenta, and the α-helix covering residues 118-149 is highlighted in yellow. Alternative conformations in PAB10Ala and PAB11Ala are colored in darker colors matching those displayed in **Fig. 1D.** Error bars were calculated based on the S/N ratio.

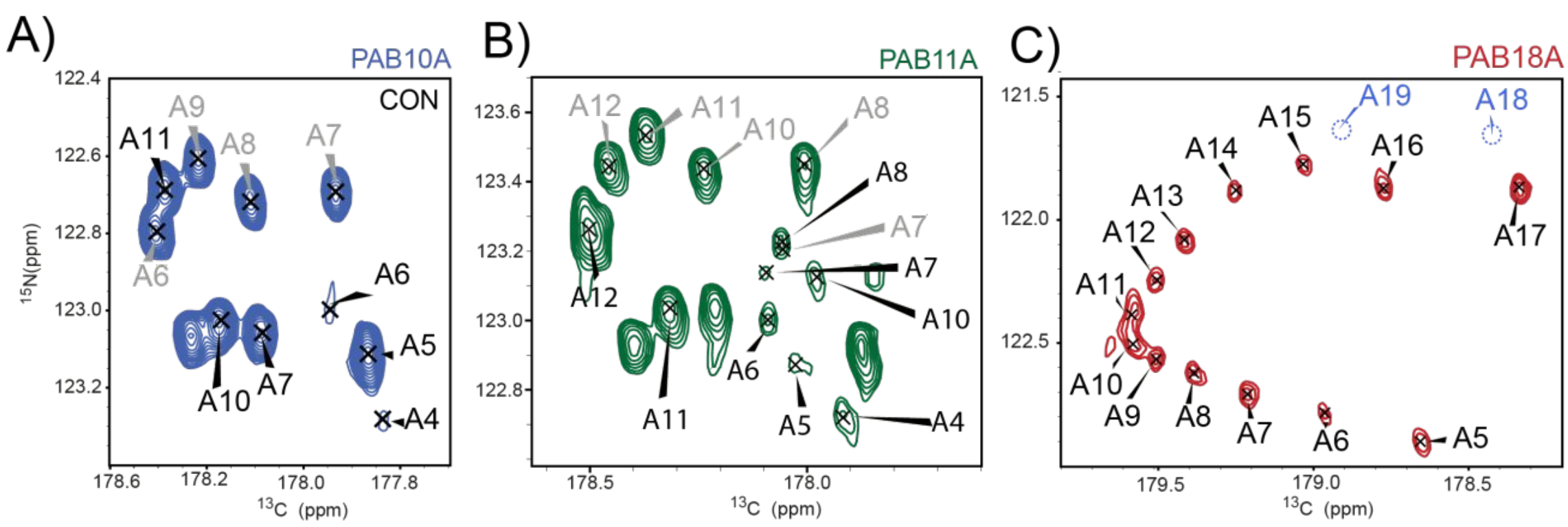

Supplementary Figure 5. Details of the CON spectra for PAB10Ala (blue), PAB11Ala (green) and PAB18Ala (red) at 25 ºC, focusing on the polyalanine segments. Assignments in grey correspond to those described in **Fig. 1C** and in **Supplementary Figures 1, 3.** Crosspeaks for Ala18 and Ala19 disappear during acquisition in PAB18Ala (panel **C**), and are located in blue circles for inspection.

Experimental Details

Sample preparation

PAB10Ala and PAB18Ala constructs covering residues 1-170 from human PABPN1 (UniProt KB Q86U42, Origene) were cloned into modified pET28a vectors (GenScript) containing a six-histidine tag for Ni^{2+} affinity purification, followed by a Tobacco Etch Virus (TEV) protease cleavage site. Polyalanine expanded PAB11Ala was generated by site-directed mutagenesis using the oligonucleotides listed below, and all sequences were verified by DNA sequencing:

	**Forward**	**Reverse**
**PAB11Ala**	5'-GCGGCAGCAGCAGCGGCAGGGGCTGCGGGCGGT-3´	5´-GACCGCCCGCAGCCCCTGCCGCTGCTGCTGCCG-3'

PABPN1 fragments (PAB10Ala/11Ala/18Ala) were produced in Rossetta 2(DE3) star *E. coli* strain. Cells were cultured in rich media until OD_{600nm}= 0.6 – 0.8. For NMR studies, proteins must be isotopically labelled with $^{15}N/^{13}C$. Once the OD is reached, cultures were harvested by a 25-minute centrifugation at 5,300 g, and the pellets resuspended in ¼ of the initial volume in M9 medium supplemented with 4g/l of ^{13}C-glucose and 1g/l of $^{15}NH_4Cl$ as the only sources of carbon and nitrogen, respectively [1]. Cultures were then incubated at 37 °C for 1–1.5 hours to allow adaptation to the new medium, until the optical density increased by one unit relative to the value measured immediately after resuspension. Protein expression, both in rich and minimal media, was induced by the addition of isopropyl-β-D-1-thiogalactopyranoside (IPTG) at a final concentration of 1 mM, followed by an incubation for 20 h at 25 ºC.

Cells were harvested by centrifugation at 5,300 g for 25 minutes at 4 °C. The resulting pellets were resuspended in 30 ml/L of lysis buffer (50 mM KPi/500 mM NaCl/10 mM imidazole/2mM β-mercaptoethanol [pH 8]). The lysate was incubated with protease inhibitor cocktail EDTA-free (Roche) and 1 mg/ml lysozyme (Sigma-Aldrich) for 30 minutes at 4 °C. Afterwards, DNase I (0,01 mg/mL), RNase A (0,01 mg/mL), Triton X-100 (0,05%), and $MgCl_2$ (5 mM) were added to the mixture followed by two incubations steps: one at 4 °C for 10 minutes and the other one for 10 minutes at room temperature (RT) with gentle agitation. The sample was then sonicated and clarified by centrifugation at 4,000 × g for 20 min at 4 °C using an Optima XPN-90 ultracentrifuge (Beckman Coulter). The soluble fraction was further incubated at RT for 40 min with RNase I (0,01 mg/mL) and $MgCl_2$ (5 mg), and finally clarified under the same centrifugation conditions to separate the supernatant from the inclusion bodies.

Proteins were present in the soluble fraction; therefore, the clarified lysate was immediately loaded into a pre-equilibrated Ni^{2+} column and eluted using an imidazole gradient until a concentration of 500 mM imidazole was reached. TEV digestion was carried out in 5 mM KPi/10 mM NaCl/10 mM Imidazole/2 mM β-mercaptoethanol [pH 8], by an overnight incubation at 4 °C with 0.5-1 mg/mL of TEV protein (produced in-house) per 15 mg of fusion protein. Cleaved PABPN1 constructs were then again applied to a Ni^{2+} affinity column and eluted under the same conditions mentioned above. Samples were stored at -80 °C and, when required, they were dialyzed in NMR buffer (5 mM KPi/10 mM NaCl/2 mM β-mercaptoethanol [pH 6.8]) and concentrated using Amicon® Ultra (Merck) ultrafiltration tubes (3 kDa MWCO).

Turbidimetry assays

Protein samples of pure PAB10Ala and PAB18Ala were prepared at a final concentration of 0.2 mM in 20 mM Tris-HCl/150 mM NaCl/2 mM DTT/1mM benzamidine/0,02% sodium azide [pH 8]

(incubation buffer). Turbidity was quantified at 25 °C by measuring absorbance at 600 nm in a CLARIOstar Plus (BMG LABTECH) multiwell plate reader using 96-well flat-bottom plates (Porvair Sciences). Samples were incubated for <40 h, with 60 s of agitation at 100 rpms before each reading, and measurements were recorded at 10-minute intervals. All data reported was technically and biologically replicated, and presented as the average between replicates with the standard deviation.

NMR experiments

All NMR spectra were acquired on a Bruker AVNEO 800 MHz (^{1}H) spectrometer equipped with a triple-resonance TCI cryoprobe and Z-gradients. NMR experiments for all PABPN1 constructs were obtained using 0.3-0.45 mM of protein concentration at 25 ºC in NMR buffer. ^{1}H chemical shifts were referenced using sodium trimethylsilyl propanesulfonate (DSS), and ^{15}N and ^{13}C chemical shifts were referenced indirectly to ^{1}H using the corresponding gyromagnetic ratios [2].

All different PABPN1 assignments were based on the acquisition of 2D ^{15}N-HSQC, 2D CON, 3D CBCA(co)NH, 3D HNCACB, 3D HNCO, and 3D HNCA NMR spectra. H(CCO)NH was acquired to confirm the identity of certain residues. Sequential connectivities were derived from high-resolution 3D HNNH experiments, which allowed unambiguous assignments of the polyalanine segments even in highly overlapped spectral regions. NMR assignments from previous spectra were used to assign the 2D CACO and 2D CON and were transferred to all different incubated spectra. Assignments in the incubated spectra were validated by acquiring 3D HNCACB, H(CCO)NH, and 3D HNNH on incubated samples. Secondary chemical shifts were calculated as $\Delta C\alpha$-$\Delta C\beta$, where $\Delta C\alpha$ and $\Delta C\beta$ represent the differences between the experimentally measured Cα and Cβ chemical shifts and the corresponding random-coil chemical shifts computed for each PABPN1 construct at a specific temperature and pH (https://spin.niddk.nih.gov/bax/nmrserver/Poulsen_rc_CS/) [3,4]. NMR spectra obtained from fresh samples revealed the presence of alanine residues populating two different conformational states. After incubation, the populations appeared to shift toward more disordered conformations. To analyse real-time changes between different conformations while minimizing spectral overlap, 2D CACO were used for PAB10Ala/11Ala, and 3D HNCO experiments were acquired for PAB18Ala for different samples, showing robust reproducibility. Heat map representations were obtained by analyzing peak intensities in the 2D CACO spectra at different time points. Intensities were normalized on a 0 to 1 scale, using an isolated, well-resolved resonance as a reference. To estimate the percentage of α-helix population in 2D CACO spectra, the difference between the Cα experimental data and the random coil chemical shift for the particular residue at a specific temperature and pH was calculated as described above [3,4]. The percentage conversion was then performed assuming that a fully formed α-helix corresponds to a secondary chemical shift of 3.1 ppm [5]. Similarly, the population of α-helix in 3D HNCO was determined using the same approach, but using the CO chemical shift deviations. In this case, the chemical shift value which correspond to a fully formed α-helix was 2.8 ppm [6].

(^{1}H)-^{15}N Heteronuclear NOE spectra were obtained using standard Bruker pulse sequences at 25 ºC. HetNOEs values were obtained from paired spectra recorded with and without proton saturation and were calculated as the ratio of peak intensities. Uncertainties in peak heights were estimated from the spectral noise. All NMR spectra were processed in Topspin 4.1.1, analyzed in Sparky 3.19 [7], and uploaded to the Biological Magnetic Resonance Data Bank (BMRB) with the codes 53584, 53591, 53594 for PAB10Ala, PAB11Ala and PAB18Ala, respectively.

References

[1] J. Marley, M. Lu, C. Bracken, "A method for efficient isotopic labeling of recombinant proteins". *J Biomol NMR.* **2001,** *20,* 71.

[2] J. L. Markley, A. Bax, Y. Arata, C. W. Hilbers, R. Kaptein, B. D. Sykes, P. E. Wright, K. Wüthrich, "Recommendations for the presentation of NMR structures of proteins and nucleic acids. IUPAC-IUBMB-IUPAB Inter-Union Task Group on the Standardization of Data Bases of Protein and Nucleic Acid Structures Determined by NMR Spectroscopy". *J Biomol NMR*. **1998,** *12,* 1.

[3] M. Kjaergaard, F. M. Poulsen, "Sequence correction of random coil chemical shifts: correlation between neighbor correction factors and changes in the Ramachandran distribution". *J Biomol NMR* **2011,** *50,* 157.

[4] M. Kjaergaard, S. Brander, F. M. Poulsen, "Random coil chemical shift for intrinsically disordered proteins: effects of temperature and pH". *J Biomol NMR* **2011,** *49,* 139.

[5] S. Spera, A. Bax,"Empirical correlation between protein backbone conformation and C-alpha and C-beta C-13 nuclear-magnetic-resonance chemical-shifts". *Journal of the American Chemical Society* **1991,** *113,* 5490–5492.

[6] D. V. Fedyukina, S. Rajagopalan, A. Sekhar, E. C. Fulmer, Y. J. Eun, S. Cavagnero, "Contribution of long-range interactions to the secondary structure of an unfolded globin". *Biophys J.* **2010,** *99,* L37.

[7] W. Lee, M. Tonelli, J. L. Markley, "NMRFAM-SPARKY: enhanced software for biomolecular NMR spectroscopy". *Bioinformatics* **2015,** *31,* 1325.
